# Complexation with Random Methyl-β-Cyclodextrin and (2-Hidroxypropyl)-β-Cyclodextrin Enhances In Vivo Anti-Fibrotic and Anti-Inflammatory Effects of Chrysin via the Inhibition of NF-κB and TGF-β1/Smad Signaling Pathways and Modulation of Hepatic Pro/Anti-Fibrotic miRNA

**DOI:** 10.3390/ijms22041869

**Published:** 2021-02-13

**Authors:** Alina Ciceu, Cornel Balta, Hidegard Herman, Sami Gharbia, Simona-Rebeca Ignat, Sorina Dinescu, Judit Váradi, Ferenc Fenyvesi, Szilvia Gyöngyösi, Anca Hermenean, Marieta Costache

**Affiliations:** 1 “Aurel Ardelean” Institute of Life Sciences, “Vasile Goldiș” Western University of Arad, 86 Revolution Str., 310048 Arad, Romania; alinaciceu80@gmail.com (A.C.); baltacornel@gmail.com (C.B.); hildegard.i.herman@gmail.com (H.H.); samithgh2@gmail.com (S.G.); 2 Department of Biochemistry and Molecular Biology, Faculty of Biology, University of Bucharest, Splaiul Independentei 91-95, 050095 Bucharest, Romania; simona.ignat@unibuc.ro (S.-R.I.); sorina.dinescu@bio.unibuc.ro (S.D.); marieta.costache@bio.unibuc.ro (M.C.); 3 Research Institute of the University of Bucharest, Splaiul Independentei 91-95, 050095 Bucharest, Romania; 4 Department of Pharmaceutical Technology, Faculty of Pharmacy, University of Debrecen, Nagyerdei St. 98, H-4032 Debrecen, Hungary; varadi.judit@pharm.unideb.hu (J.V.); fenyvesi.ferenc@pharm.unideb.hu (F.F.); 5 Department of Solid State Physics, University of Debrecen, P.O. Box 400, H-4002 Debrecen, Hungary; gyongyosi.szilvia@science.unideb.hu; 6 Department of Histology, Faculty of Medicine, Vasile Goldiș Western University of Arad, 86 Revolution Str., 310414 Arad, Romania

**Keywords:** chrysin, HPBCD, RAMEB, liver, fibrosis, inflammation

## Abstract

Chrysin (CHR) is a natural flavonoid with a wide range of pharmacological activities, including hepatoprotection, but poor water solubility. By including water-soluble hydroxypropyl (HPBCD) and randomly methylated (RAMEB) β-cyclodextrin, we aimed to increase its biodisponibility and the effectiveness of the antifibrotic effects of chrysin at oral administration. Liver fibrosis in mice was induced in 7 weeks by CCl_4_ i.p. administration, and afterwards treated with 50 mg/kg of CHR-HPBCD, CHR-RAMEB, and free chrysin. CCl_4_ administration increased hepatic inflammation (which was augmented by the upregulation of nuclear factor kappa-light-chain enhancer of activated B cells (NF-kB), tumor necrosis factor (TNF)-α, and interleukin 6 (IL-6) and induced fibrosis, as determined using histopathology and electron microscopy. These results were also confirmed by the upregulation of Collagen I (Col I) and matrix metalloproteinase (MMP) expression, which led to extracellular fibrotic matrix proliferation. Moreover, the immunopositivity of alpha-smooth muscle actin (a-SMA) in the CCl_4_ group was evidence of hepatic stellate cell (HSC) activation. The main profibrotic pathway was activated, as confirmed by an increase in the transforming growth factor- β1 (TGF-β1) and Smad 2/3 expression, while Smad 7 expression was decreased. Treatment with CHR–HPBCD and CHR–RAMEB considerably reduced liver injury, attenuated inflammation, and decreased extracellular liver collagen deposits. CHR–RAMEB was determined to be the most active antifibrotic complex. We conclude that both nanocomplexes exert anti-inflammatory effects and antifibrotic effects in a considerably stronger manner than for free chrysin administration.

## 1. Introduction

Liver fibrosis is a healing response to extensive or prolonged injuries induced by alcohol abuse [1], xenobiotics, chronic viral hepatitis [2], cholestasis, or autoimmune liver diseases [3] with extensive extracellular matrix (ECM) deposition [4]. Parenchymal scars, which are mainly composed of Type I and III collagens, are initially deposited in portal triads and/or in the parenchyma, depending on the injured structure [5]. Subsequently, morphological and functional changes may progress to cirrhosis, which is characterized by fibrotic nodules and by a decrease in hepatic blood supply [6].

ECM production in the injured liver is attributed to hepatic stellate cells (HSCs). In a quiescent state, HSCs localized in the space of Disse are responsible for Vitamin A storage. During activation, they start expressing alpha-smooth muscle actin (a-SMA) and synthesize an extracellular matrix [7]. The progression of fibrogenesis depends on activated myofibroblasts originating from liver-resident HSCs, from epithelial cells by epithelial–mesenchymal transition (EMT), and less from hepatocytes or bone marrow (BM)-derived cells [8,9]. Myofibroblasts are the main effectors involved in fibrogenesis owing to excessive collagen production and unbalanced extracellular matrix (ECM) deposition [10].

Fibrogenesis promotes the release of inflammatory cytokines (e.g., IL-17, -22, and -33) by immune cells (including Kupffer cells [8] and chemokines (CCL2) involved in the recruitment of leukocytes into injured liver [11]) and the release of growth factors (e.g., transforming growth factor (TGF) and platelet-derived growth factor (PDGF) [12]. Transforming growth factor-β (TGF-β) is essential in liver fibrogenesis [13]. TGF-β is released by Kupffer cells and can also be secreted by activated HSCs, and is responsible for the activation and trans-differentiation of quiescent hepatic stellate cells (HSCs) and fibroblasts [10] into a contractile myofibroblast phenotype, which expresses α-SMA, promotes epithelial mesenchymal transition (EMT) and the recruitment of immune cells, and finally induces the synthesis of ECM proteins [14].

TGF-β binds to Type I and II receptor complexes, followed by the activation of canonical Smad-dependent signaling pathways. In addition, TGF-β can use Smad-independent pathways, including non-receptor tyrosine kinase proteins (e.g., Src or FAK), nuclear factor kappa-light-chain enhancer of activated B cells (NF-kB) and PI3K/Akt pathways, which can also regulate the canonical Smad pathway and affect biological responses mediated by TGF-β [14].

Matrix metalloproteinases (MMPs) are a family of zinc-dependent enzymes that degrade ECM proteins. According to their substrate specificity, five major groups are known: collagenases, gelatinases, membrane-type, stromelysins, and matrilysins [15]. The proteolytic activities of MMPs in tissues are regulated by tissue inhibitors of metalloproteinases (TIMPs), which is a family of four inhibitors (TIMP 1–4) [15,16]. The dysregulation of MMPs and unbalanced MMPs/TIMP activity result in structural and functional changes and ECM deposition.

MicroRNAs (miRNAs) have emerged as a class of small non-coding RNAs that regulate gene expression at post-transcriptional level. Several studies have evaluated the role of miRNAs in liver fibrosis, and the obtained results showed upregulated and downregulated miRNAs in liver fibrosis models [17,18,19,20]. Further studies identified the specific roles of miRNAs as pro- or antifibrotic [21]. MiR-29 is considered to be the main antifibrotic regulator, which is also associated with low levels of fibrosis in other organs. MiR-29 is involved in the post-translational processing of ECM, especially in collagen Type I downregulation. Furthermore, miR-29 can induce the apoptosis of activated HSCs, which reduces the number of collagen-producing cells. Other antifibrotic miRNAs identified in previous studies are miR-192, -193, -194, -16, -378, and -878 [19,22]. Of these, miR-192 and -194 are specifically involved in attenuating HSC activation, migration, and proliferation [18,23]. Profibrotic miRNAs involved in HSC activation and ECM formation are miR-138, -146b, -147, -199a, -34a, and -466l [17,18,19,20]. MiR-34a promotes liver fibrosis by mediating the Sirt1/p53 signaling pathway [24].

Promising antifibrotic therapies are in different research stages; some therapies are even being clinically validated and are based on the inhibition of profibrogenic factors or pathways. The potential targets of the different treatment strategies involve different pathways related to immune cells, cytokines (including activated growth factor), oxidative stress, or nuclear receptor signaling [25].

Chrysin is a natural flavonoid that has been widely used in traditional medicine and has been reported to exhibit a wide range of pharmacological activities. Chrysin exhibits hepatoprotective activity against drugs or chemical compounds [26]. Moreover, it ameliorates metabolically induced hepatic steatosis [27] and non-alcoholic fatty liver disease in rats [28]; our previous study experimentally showed the antifibrotic action of chrysin in a fibrosis model in mice induced by CCl_4_ [29,30].

In our previous study, we developed a novel drug delivery system of chrysin in randomly methylated β-cyclodextrin (RAMEB) and hydroxypropyl β-cyclodextrin (HPBCD) cyclodextrins [31], and we showed its hepatoprotective effects in vitro [32]. In this research, we aimed to evaluate the efficacy of chrysin–RAMEB/HPBCD nanocomplexes at reversing hepatic fibrosis in vivo in a mouse model of CCl_4_-induced hepatic fibrosis in a more efficient way than pure chrysin.

## 2. Results

### 2.1. Characterization of CHR-HPBCD and CHR-RAMEB Nanocomplexes

**Figure 1:**
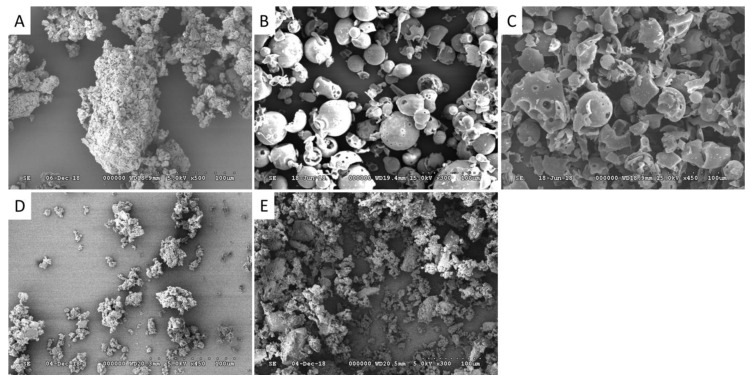
SEM images of raw chrysin (**A**), hydroxypropyl β-cyclodextrin (HPBCD) (**B**), randomly methylated β-cyclodextrin (RAMEB) (**C**), the chrysin–HPBCD complex (**D**), and the chrysin–RAMEB complex (**E**).

The morphology of raw chrysin and chrysin–cyclodextrin complexes was examined by SEM (Figure 1). Raw chrysin exhibited aggregated particles with various shapes and a wide particle size distribution. Electron microscopic analysis showed that HPBCD particles have a spherical and intact shape, while those of RAMEB are mostly broken. Chrysin–HPBCD or RAMEB complexes exhibited a completely different morphology. After the inclusion process, the original cyclodextrin and chrysin particles were unidentifiable; however, the aggregates containing smaller amorphous particles revealed the interaction between chrysin and cyclodextrins. The formed complexes contribute to the higher solubility and improved bioavailability of chrysin.

### 2.2. CHR–HPBCD and CHR–RAMEB Nanocomplexes Alleviate CCl_4_-Induced Liver Fibrosis Collagen Deposition, and Ultrastructural Changes

**Figure 2:**
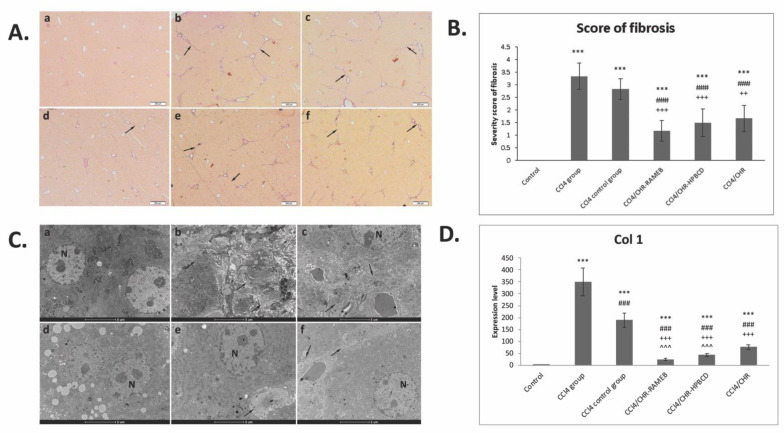
Effect induced by chrysin (CHR)–HPBCD and CHR–RAMEB complexes on liver fibrosis resolution (**A**) Fouchet van Gieson trichrome staining. (a) Control group: no significant collagen deposition; (b) CCl_4_ group: significant collagen deposition with large fibrous septa formation and pseudo-lobular separation; (c) CCl_4_ control group: aspect almost similar to the CCl_4_ group; (d) CCl_4_/CHR–RAMEB co-treated group: less collagen deposition compared with the CCl_4_ control; (e) CCl_4_/CHR–HPBCD co-treated group: histological aspect closest to the control; (f) CCl_4_/CHR co-treated group: fibrotic changes are still present; arrow—fibrotic septa and collagen deposition. Scale bar: 50 μm. (**B**) Histogram showing the fibrosis score in trichrome stain. (**C**) Collagen deposition in livers by transmission electron microscopy (TEM): (a) Control group: normal ultrastructure of hepatocytes; (b) CCl_4_ group: proliferation of collagen into the parenchyma; (c) CCl_4_ control group: the ultrastructural recovery is weak and parenchymal collagen was noticed; (d) CCl_4_/CHR-RAMEB co-treated group: ultrastructural aspect closest to the control; (e) CCl_4_/CHR-HPBCD co-treated group: parenchymal collagen areas are still present; (f) CCl_4_/CHR co-treated group: fibrotic changes are still present (**D**). RT-PCR analysis of Collagen 1 (Col 1) gene levels. *** *p* < 0.001 compared with the control; ^###^ *p* < 0.001 compared with the CCl_4_ group; ^+++^ *p* < 0.001 compared with the CCl_4_ control group; ^++^ *p* < 0.01 compared with the CCl_4_ control group; ^^^ *p* < 0.001 compared with the CCl_4_/CHR group.

Liver fibrosis was analyzed histologically using Fouchet van Gieson’s trichrome stain (Figure 2A). The control liver has a normal lobular structure without any proliferation of collagen. The liver histology of the CCl_4_ group showed severe changes such as collagen deposits and formation of pseudo-lobules, and the fibrosis score increased compared with the control (*p* < 0.001). Moreover, electron microscopy micrographs showed collagen infiltration into liver parenchyma and hepatocyte alterations (Figure 2C). The mRNA expression of Collagen I (Col I) was also considerably increased in the fibrotic group compared with the control (*p* < 0.001) (Figure 2D). The extent of fibrotic change was still noticed in the CCl_4_ control group (*p* < 0.001) compared with that in the control. Treatment with CHR–HPBCD or CHR–RAMEB considerably reduced the score of liver fibrosis compared with the CCl_4_ group (*p* < 0.001). The fibrosis score and thickness of fibrous septa were considerably decreased in the CHR–RAMEB group compared with the CHR–HPBCD group (*p* < 0.05), whereas the free chrysin group exhibited a significantly increased fibrosis score compared with those treated with chrysin complexes (Figure 2B).

### 2.3. CHR–HPBCD and CHR–RAMEB Nanocomplexes Inhibit the Activation of Hepatic Stellate Cells 

HSCs play a major role in the progression of hepatic fibrosis [33]. In an injured condition they are activated, proliferate, and have the ability to trans-differentiate into myofibroblast-like cells which produce large amounts of ECM components and collagen. α-SMA is a good marker for the detection of activated HSCs during fibrogenesis; therefore, α-SMA-immunoreactive cells are increased in number and reactivity in liver fibrosis [34,35].

**Figure 3:**
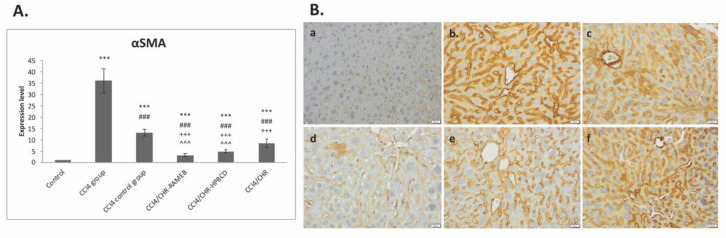
Effects of CHR–HPBCD and CHR-RAMEB complexes on α-SMA induced hepatic stellate cell (HSC) activation. (**A**) RT-PCR analysis of α-SMA gene levels. *** *p* < 0.001 compared with the control; ^###^ *p* < 0.001 compared with the CCl_4_ group; ^+++^ *p* < 0.001 compared with the CCl_4_ control group; ^^^ *p* < 0.001 compared with the CCl_4_/CHR group. (**B**) Immunohistochemical expression of α-SMA. (a) Control group; (b) CCl_4_ group; (c) CCl_4_ control group; (d) CCl_4_/CHR–RAMEB co-treated group; (e) CCl_4_/CHR–HPBCD co-treated group; (f) CCl_4_/CHR co-treated group. Scale bar: 20 μm.

The impact of the CHR–HPBCD/RAMEB treatment on the regulation of α-SMA, a marker of activated HSCs, was assessed by immunohistochemistry and mRNA analysis. Figure 3B shows that the administration of carbon tetrachloride caused a considerable increase in immunopositivity for α-SMA, and it remained at high levels 2 weeks after the discontinuation of the treatment. The treatments decreased the number of cells labeled with the α-SMA antibody, especially for CHR–RAMEB, compared with the CCl_4_ group. Moreover, these results were confirmed by α-SMA downregulation (Figure 3A). Specifically, for inclusion groups, the mRNA level was considerably reduced by approximately 19.96-fold (CHR–HPBCD), 10.41-fold (CHR–RAMEB), and 7.61-fold (CHR) compared with the corresponding levels in the fibrotic livers; the reduction in the CCl_4_ control group was only 4.22-fold (Figure 3A).

### 2.4. CHR–HPBCD and CHR–RAMEB Complexes Downregulate the TGF-β1/Smad Signaling Pathway

TGF-β1 is considered to be an essential component promoted in liver fibrosis pathogenesis; TGF-β1 acts through Smad 2/3 phosphorylation. Furthermore, Smad 7 is a Smad inhibitor; it acts through Smad 2/3 downregulation and acts by targeting the TGF-β1 receptor [36].

**Figure 4:**
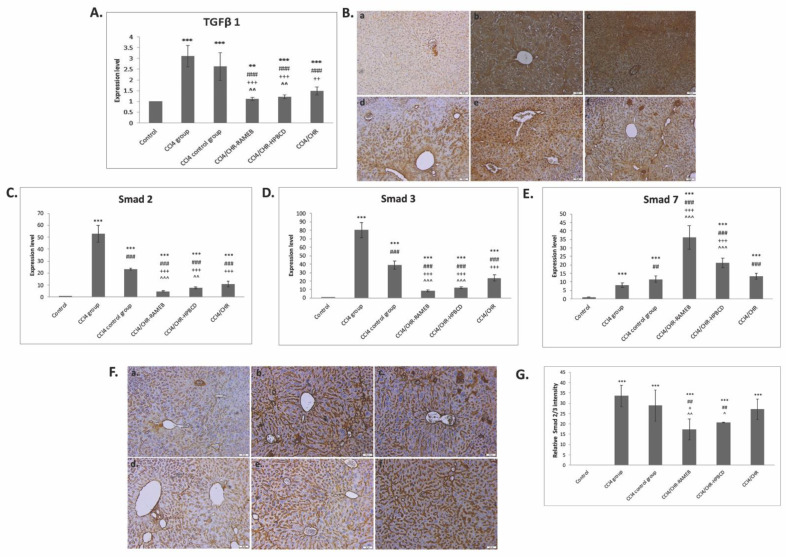
Effects of CHR–HPBCD and CHR–RAMEB nanocomplexes on transforming growth factor- β1 (TGF-β1)/Smad signalling pathway downregulation. (**A**) RT-PCR analysis of TGF-β1 gene expression levels. (**B**) Immunohistochemical expression of TGF-β1. (a) Control group; (b) CCl_4_ group; (c) CCl_4_ control group; (d) CCl_4_/CHR–RAMEB co-treated group; (e) CCl_4_/CHR–HPBCD co-treated group; (f) CCl_4_/CHR co-treated group. Scale bar: 50 μm; (**C**) RT-PCR analysis of Smad2 gene expression levels. (**D**) RT-PCR analysis of Smad3 gene expression levels. (**E**) RT-PCR analysis of Smad7 gene expression levels. (**F**) Immunohistochemical expression of TGF-β1 (a) Control group; (b) CCl_4_ group; (c) CCl_4_ control group; (d) CCl_4_/CHR–RAMEB co-treated group; (e) CCl_4_/CHR–HPBCD co-treated group; (f) CCl_4_/CHR co-treated group. Scale bar: 50 μm. (**G**) Quantification of tumor necrosis factor (TNF)-α relative staining intensity to control and experimental groups. Results are represented as the mean ± standard deviation. *** *p* < 0.001 compared with the control; ** *p* < 0.01 compared with the control; ^###^ *p* < 0.001 compared with the CCl_4_ group; ^##^ *p* < 0.01 compared with the CCl_4_ group; ^+++^ *p* < 0.001 compared with the CCl_4_ control group; ^++^ *p* < 0.01 compared with the CCl_4_ control group; ^+^ *p* < 0.05 compared with the CCl_4_ control group; ^^^ *p* < 0.001 compared with the CCl_4_/CHR group; ^^ *p* < 0.01 compared with the CCl_4_/CHR group; ^ *p* < 0.05 compared with the CCl_4_/CHR group.

Compared with the control, CCl_4_-induced liver fibrosis was associated with a considerable upregulation of TGF-β1 gene expression and strong immunopositivity (Figure 4A,B). TGF-β1 was considerably reduced by CHR–RAMEB, CHR–HPBCD, and CHR treatments by 2.77-, 2.55-, and 2.08-fold, respectively, compared with the CCl_4_ fibrotic group, and the spontaneous reversal of fibrosis (Group 3) was lower compared with that in all treatment groups.

The Smad signaling pathway is essential for transmitting TGF-β1 superfamily signals from the cell surface to the nucleus. The hepatic mRNA levels of Smad2 in the CHR–RAMEB, CHR–HPBCD, and CHR groups were considerably reduced by approximately 11.15-, 7.01-, and 4.96-fold, respectively, compared with the corresponding levels in the CCl_4_ fibrotic group, whereas the reduction was 1.18-fold for the CCl_4_ control group (Figure 4C). The results were similar for the mRNA levels of Smad3 (Figure 4D). In contrast, the CHR–RAMEB and CHR–HPBCD treatments considerably increased the expression of Smad7 compared with that in the fibrotic group (*p* < 0.001); the increase was less significant for the group with spontaneous fibrosis resolution (*p* < 0.01) (Figure 4E).

### 2.5. CHR–HPBCD and CHR–RAMEB Nanocomplexes Downregulate the NF-kB-Mediated Inflammatory Pathway

NF-κB is a key transcriptional regulator of the inflammatory response and plays an essential role in the regulation of inflammatory signaling pathways in the liver, and is an integral part of the hepatic wound-healing response to injury [37,38]. In order to be active, NF-κB needs to be translocate to the nucleus and initiate gene transcription, further stimulate Toll-like receptors (TLRs), as well as inflammatory cytokines such as tumor necrosis factor (TNF) or interleukins [37]. Therefore, we aimed to highlight the ability of chrysin–cyclodextrin complexes to inhibit NF-κB translocation and to block the overproduction of proinflammatory cytokines.

**Figure 5:**
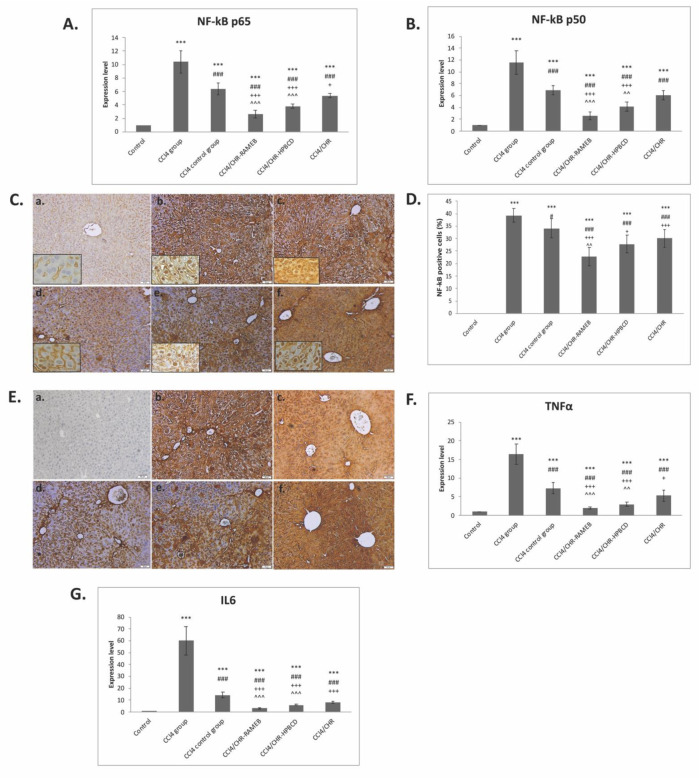
Effects of CHR–HPBCD and CHR–RAMEB nanocomplexes on NF-kB-mediated inflammatory pathway down-regulation. (**A**) RT-PCR analysis of NF-kB p50 gene expression levels. (**B**) RT-PCR analysis of NF-kB p65 gene expression levels. (**C**) Immunohistochemical expression of NF-kB p65: (a) Control group; (b) CCl_4_ group; (c) CCl_4_ control group; (d) CCl_4_/CHR–RAMEB co-treated group; (e) CCl_4_/CHR–HPBCD co-treated group; (f) CCl_4_/CHR co-treated group. Scale bar: 50 μm. (**D**) The percentage of NF-κB p65-positive cells in three representative fields. (**E**) Immunohistochemical expression of TNF-α: (a) Control group; (b) CCl_4_ group; (c) CCl_4_ control group; (d) CCl_4_/CHR–RAMEB co-treated group; (e) CCl_4_/CHR–HPBCD co-treated group; (f) CCl_4_/CHR co-treated group. Scale bar: 50 μm. (**F**) RT-PCR analysis of TNF-α gene expression levels. (**G**) RT-PCR analysis of IL-6 gene expression levels. *** *p* < 0.001 compared with the control; ^###^ *p* < 0.001 compared with the CCl_4_ group; ^#^ *p* < 0.05 compared with the CCl_4_ group; ^+++^ *p* < 0.001 compared with the CCl_4_ control group; ^+^ *p* < 0.05 compared with the CCl_4_ control group; ^^^ *p* < 0.001 compared with the CCl_4_/CHR group; ^^ *p* < 0.01 compared with the CCl_4_/CHR group.

A significant increase in NF-kB, TNF-α, and IL-6 gene expression was detected in fibrotic livers compared with the control (Figure 5A,B,F,G). Two weeks of daily CHR–RAMEB or CHR–HPBCD oral administration induced a significant downregulation of all genes compared with the CCl_4_ group (*p* < 0.001). The anti-inflammatory activity of CHR–RAMEB was more pronounced. Therefore, the immunopositivity of NF-kB and TNF-α has the same pattern as the gene expression level (Figure 5C). The percentage of NF-κB positive cells of CHR–RAMEB or CHR–HPBCD were significantly decreased compared with the fibrotic groups (*p* < 0.001) (Figure 5D).

### 2.6. CHR–HPBCD and CHR–RAMEB Nanocomplexes Modulate ECM by TIMP-1/MMPs Balance

**Figure 6:**
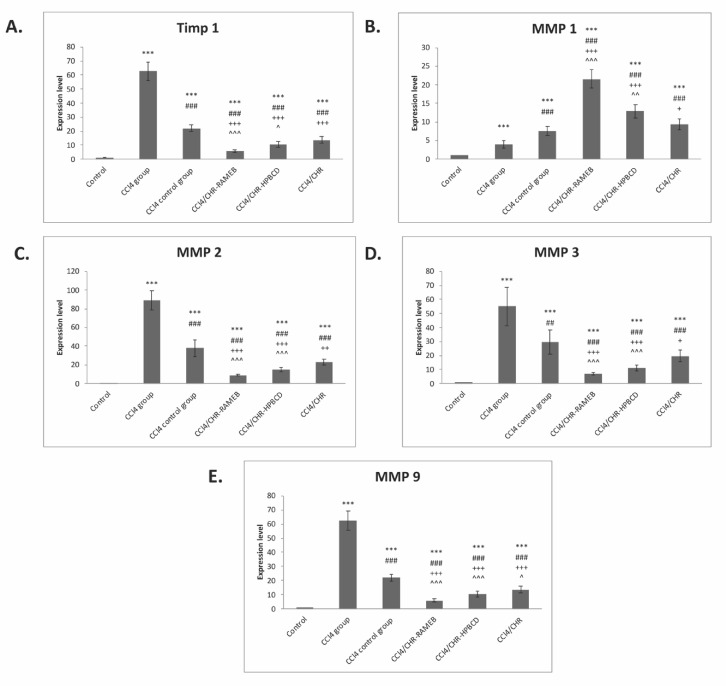
Effects of CHR–HPBCD and CHR–RAMEB nanocomplexes on tissue inhibitors of metalloproteinase (TIMP)-1/matrix metalloproteinase (MMP) pathway regulation. (**A**) RT-PCR analysis of Timp1 gene expression levels. (**B**) RT-PCR analysis of MMP1 gene expression levels. (**C**) RT-PCR analysis of MMP2 gene expression levels. (**D**) RT-PCR analysis of MMP3 gene expression levels. (**E**) RT-PCR analysis of MMP9 gene expression levels. *** *p* < 0.001 compared with the control; ^###^ *p* < 0.001 compared with the CCl_4_ group; ^##^ *p* < 0.01 compared with the CCl_4_ group; ^+++^ *p* < 0.001 compared with the CCl_4_ control group; ^++^ *p* < 0.01 compared with the CCl_4_ control group; ^+^ *p* < 0.05 compared with the CCl_4_ control group; ^^^ *p* < 0.001 compared with the CCl_4_/CHR group; ^^ *p* < 0.01 compared with the CCl_4_/CHR group; ^ *p* < 0.05 compared with the CCl_4_/CHR group.

Hepatic ECM deposition is especially balanced by TIMP-1, which is an inhibitor of the MMPs degradation of matrix components. To investigate the inhibitory effects of CHR–RAMEB and CHR-–HPBCD on ECM deposition in fibrotic livers, the mRNA levels of TIMP-1 and MMP-1, 2, 3, and 9 were measured by RT-PCR analysis (Figure 6). Our data showed that the expression levels of these genes were considerably increased (*p* < 0.001) in the CCl_4_ fibrotic group compared with the control. Treatment with CHR–RAMEB or CHR–HPBCD considerably downregulated the mRNA levels of MMP-2, MMP-3, MMP-9, and TIMP-1 compared with both the CCl_4_ and CCl_4_ control groups (*p* < 0.001). In contrast, the mRNA expression of MMP-1 was considerably higher for the CHR–RAMEB and CHR–HPBCD treated groups compared to those of the CCl_4_ and CCl_4_ control groups (*p* < 0.001).

### 2.7. CHR–HPBCD and CHR–RAMEB Nanocomplexes Modulate Profibrotic and Antifibrotic miRNA Expression

**Figure 7:**
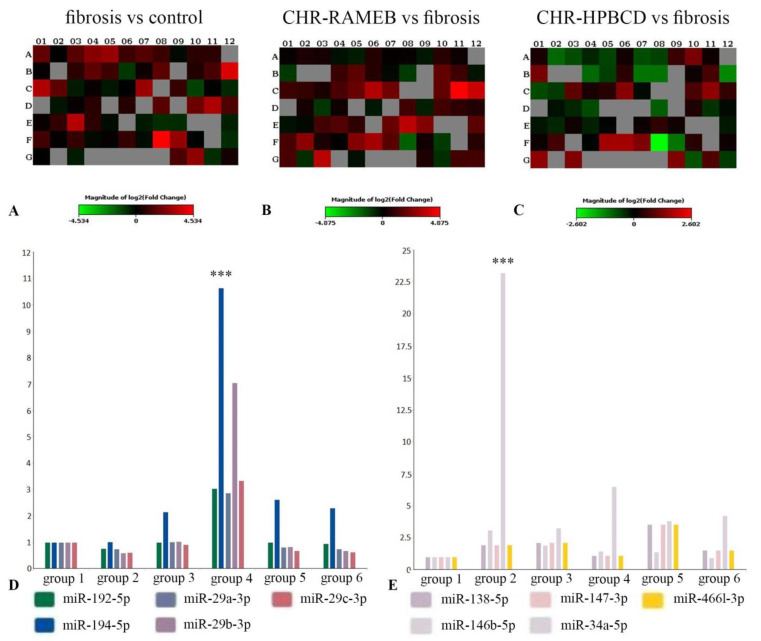
Effects of CHR-HPBCD and CHR-RAMEB nanocomplexes on miRNA expression regulation. (**A**) Heat map of the fold change between the CCl_4_ group and the control group. (**B**) Heat map of the fold change between the CCl_4_/CHR-RAMEB co-treated group and the CCl_4_ group. (**C**) Heat map of fold change between the CCl_4_/CHR-HPBCD co-treated group and the CCl_4_ group. (**D**) Multigroup chart of antifibrotic miRNA expression (miR-192-5p, miR-194-5p, miR-29a-3p, miR-29b-3p, and miR-29c-3p) in all groups tested, *** *p* < 0.001 (Group 4 vs. Groups 2 and 5). (**E**) Multigroup chart of profibrotic miRNAs expression (miR-138-5p, miR-146b-5p; miR-147-3p, miR-34a-5p, and miR-466l-3p) in all groups tested, *p* < 0.001 (Group 2 vs. Groups 4 and 5). Group 1-Control group; Group 2-CCl_4_ group; Group 3-CCl_4_ control group; Group 4-CCl_4_/CHR–RAMEB co-treated group; Group 5-CCl_4_/CHR–HPBCD co-treated group; Group 6-CCl_4_/CHR co-treated group.

The expression of 84 fibrosis-associated miRNAs from liver samples was evaluated by qPCR using a miScript miRNA PCR array (Figure 7). The fold change regulation expression between different groups is graphically represented as heat maps in Figure 7A–C. The expression of 27 miRNAs was determined to be upregulated in the CCl_4_ group compared with the control group, and two miRNAs were downregulated (Figure 7A). The treatment with CHR incorporated in RAMEB and HPBCD modulated the expression of miRNAs compared with the CCl_4_ group (Figure 7B,C). The expression of antifibrotic miRNAs (miR-192-5p, miR-194-5p, miR-29a-3p, miR-29b-3p, and miR-29c-3p) was observed in all six tested groups (Figure 7D). As a result of the CCl_4_ treatment, the expression of antifibrotic miRNAs was either downregulated or remained at the same levels as in the control. For the CCl_4_/CHR–RAMEB co-treated group, the expression of antifibrotic miRNAs was considerably upregulated compared with the CCl_4_ group, which indicated the positive influence of CHR. Treatment with CHR–HPBCD stimulated the expression of antifibrotic miRNAs but at similar levels to the CCl_4_ control and CHR groups. This result suggested the superior ability of RAMEB compared with HPBCD to release CHR, which further considerably upregulated the expression of miRNAs that are responsible for liver fibrosis reversion. The same superiority of CHR–RAMEB was also observed for the expression of profibrotic miRNAs (Figure 7E). The expression of profibrotic miRNAs (miR-138-5p, miR-146b-5p; miR-147-3p, miR-34a-5p, and miR-466l-3p) was upregulated in the liver fibrosis mouse model. The expression of profibrotic miRNAs (miR-138-5p, miR-146b-5p; miR-147-3p, and miR-466l-3p) was about three times higher in the CCl_4_/CHR–HPBCD co-treated group compared with the CCl_4_/CHR–RAMEB co-treated group. This result suggested that the CHR–RAMEB treatment was better at reducing the expression of profibrotic miRNAs involved in promoting liver fibrosis.

## 3. Discussion

The oral bioavailability of chrysin is reduced owing to its poor aqueous solubility, which results in its limited efficacy in vivo and limited medical application. However, it is necessary to develop new products to improve the solubility of chrysin while maintaining its biosorption efficacy in the intestinal lumen and its stability against enzymatic hydrolysis, and to induce sustainable release [39].

To address this issue, we developed a novel drug delivery system of chrysin in cyclodextrins, such as RAMEB and HPBCD [31], which have shown their hepatoprotective effects in vitro [32]. Furthermore, in this study, we aimed to evaluate the efficacy of including nanocomplexes to reverse hepatic fibrosis in vivo in a mouse model of CCl_4_-induced hepatic fibrosis.

Liver fibrosis is a healing response to injuries; it is a dynamic interaction between cellular and molecular processes, which lead to an imbalance between ECM synthesis and degradation; therefore, progressive architectural tissue remodeling will occur and characteristic lobular scars will form that will functionally alter the liver [40]. In this study, we showed that the fibrosis score considerably decreased in fibrotic livers after 2 weeks of oral administration of CHR–RAMEB/HPBCD compared with the fibrosis control group and to the free chrysin group. In addition, these results were complemented by ultrastructural observations and the gene expression of tissue collagen, which is consistent with our previous results [29,30,41].

HSCs are non-parenchymal cells that are localized in the perisinusoidal space of Disse; they are activated during fibrogenesis by paracrine signals from resident and inflammatory cells [42]. They are the main source of matrix-producing myofibroblasts, and they are essential for the initiation and progression of liver fibrosis; therefore, preventing their activation can decrease the deposition of ECM components and alleviate or even reverse liver fibrosis. The expression of α-SMA is the most prominent marker for activated HSCs, which drive cellular motility and contraction [43]. Our results showed that α-SMA gene expression and hepatic distribution was considerably increased in fibrotic livers, which is consistent with previous studies on liver fibrosis [29]. Meanwhile, treatment by CHR–HPBCD and CHR–RAMEB considerably decreased α-SMA gene expression in CCl_4_-induced liver fibrosis mice, whereas free chrysin had lower protection (Figure 3).

TGF-β signaling was determined to be the main effective target of hepatic fibrosis because its activation leads to the differentiation of fibroblasts into contractile myofibroblasts, increased expression of α-SMA, and synthesis of extracellular matrix proteins. In this study, both chrysin–cyclodextrin inclusions considerably alleviated the mRNA expression of TGF-β1, which demonstrated their inhibitory activity against the enhanced activity of HSCs, which was confirmed by lower collagen synthesis and tissue deposition compared with fibrotic livers even after 14 days of recovery (Figure 4). Moreover, liver fibrosis was associated with an imbalance in Smad signaling by the upregulation of Smad 2 and 3 and not of Smad 7 (Figure 4), which contributed to the progression of liver fibrosis. Our results showed that both CHR–RAMEB/HPBCD treatments were able to downregulate the expression of Smad 2/3 and block the inhibitory effect of CCl_4_ on Smad 7 expression, which could contribute to the rebalancing of TGF-β/Smad signaling [44]. The modulation was more pronounced for chrysin–cyclodextrin complexes than for the non-complexed flavonoid. Therefore, we hypothesized that decreased fibrosis levels after CHR–HPBCD and CHR–RAMEB treatment could be mediated by inhibition of the TGF-β1/Smad signaling pathway, as we have previously shown for pure chrysin [29].

MMPs regulate collagen and other extracellular matrix protein levels by proteolysis and are, in turn, regulated by TIMPs. To determine whether chrysin inclusion complexes affected the expression of MMPs and TIMPs, RT-PCR was performed (Figure 6). Treatment with CCl_4_ caused an increase in the expression of TIMP1 and a decrease in the expression of MMP1, and could activate latent TGF-β, which is the major profibrotic cytokine [45]. Furthermore, CHR–RAMEB/HPBCD showed the ability to rebalance the protein level of MMPs and TIMP-1. The expression of MMP2, MMP3, and MMP9 was increased by the CCl_4_ treatment and lowered by the CHR complexes. This study found that chrysin/cyclodextrin complexes were able to upregulate MMP-1 gene expression and further stimulate cleavage of the native fibrillar collagens, especially Col I, by regulating the ECM balance via TIMP-1/MMP-1 components. MMP-1 was downregulated in fibrotic livers, but was expressed again during the resolution process, and may act through ECM degradation as well, confirmed by previous findings [30,46,47].

The nuclear factor kappa-light-chain enhancer of activated B cells (NF-kB) is an essential transcription factor that is involved in chronic inflammation and liver fibrosis [48]. NF-kB p50 activates transcription when it forms heterodimers with subunits that contain transactivation domains, especially p65 [49]. TNF-α can induce MMP9 synthesis through TNFR1 in an NF-κB-dependent manner, which may contribute to the remodeling of ECM [50] and may enhance HSC survival [51]. Our results suggest that CHR–HPBCD and CHR–RAMEB complexes are essential for modulation of the immune response and the production of extracellular matrix protein rebalancing by the downregulation of TNF-α and IL-6 through NF-kB regulatory gene inhibition.

The miRNA expression profile analysis showed that profibrotic miRNAs were upregulated in the CCl_4_ group, and antifibrotic miRNAs were either downregulated or maintained at a level similar to that of the control; these results are consistent with those reported in other studies [17,18,19,20] (Figure 7). Co-treatment with CCl_4_/CHR–RAMEB considerably modulated the expression of anti- and profibrotic miRNAs compared with the CCl_4_ group, which suggested that the treatment promoted liver fibrosis reversion at the post-transcriptional level. CHR–RAMEB treatment considerably upregulated the expression of antifibrotic miRNAs (miR-192-5p, miR-194-5p, miR-29a-3p, miR-29b-3p, and miR-29c-3p) compared with CHR–HPBCD. Of note, the expression of the master regulator miR-29 family was considerably enhanced by CHR-RAMEB treatment compared with the CHR-HPBCD. Because miR-29 is involved in reducing collagen Type I production, the low expression of collagen Type I (Figure 2D) can be correlated with the high expression of miR-29a/b/c in the CHR-RAMEB-treated group (Figure 2D). In addition, the low expression of α-SMA, which is a marker of HSC activation (Figure 2A), in co-treated CCl_4_/CHR–RAMEB, could be the result of the action of highly expressed miR-194, which inhibits HSC activation. For profibrotic miRNAs (miR-138-5p, miR-146b-5p, miR-147-3p, miR-34a-5p, miR-466l-3p), CHR-RAMEB considerably downregulated their expression compared with CHR–HPBCD. This result suggested that RAMEB was possibly better at releasing CHR than HPBCD, and that CHR was able to further stimulate the expression of antifibrotic miRNAs and inhibit profibrotic miRNAs.

Liver fibrosis is a complex pathology that is the result of multiple dysregulated pathways, including pathways that regulate inflammation and matrix secretion. Pathways such as TGFβ and NF-kB involved in liver fibrosis development are potentially regulated by miRNAs. There are a number of miRNAs that were extensively studied and were shown to be directly involved in TGF-β expression, by acting upon TGF-β receptors (miR-21, the miR-17/92 cluster, miR-106b, miR-211, miR-590, and miR-199a), TGF-β signaling components (miR-200 family, miR-182, and miR-192), and downstream TGF-β target genes (miR-106b/205 cluster, miR-17/92 cluster, and miR-146b) [52,53,54]. Some of these miRNAs were included in our study as well, such as the antifibrotic miRNAs (miR-192 and miR-199a) and the profibrotic miR-146b, and others as well that were not specifically detailed. MiRNAs such as miR-378 also promote hepatic inflammation via modulation of the NF-kB pathway [55]. However, the interplay among the TGFβ pathway, the NF-kB pathway, and the miRNA control is not completely elucidated and could represent a future research subject in itself.

## 4. Materials and Methods

### 4.1. Preparation of Chrysin-Loaded HPBCD/RAMEB Nanocomplexes

Chrysin–cyclodextrin complexes were produced with HPBCD and RAMEB (CycloLab, Budapest, Hungary), as previously described [31]. The complexes were obtained by the solvent evaporation method. Chrysin (Merck, Darmstadt, Germany) was dissolved first in 96% ethanol to obtain a 4 mg/mL chrysin solution, ed by RAMEB and HPBCD dissolution in the ethanolic chrysin solution. Cyclodextrins and chrysin were concentrated and dried during ethanol evaporation at 30 °C, and ground under continuous mixing.

### 4.2. Scanning Electron Microscopy

The morphology of RAMEB, HPBCD, chrysin, and chrysin–cyclodextrin complexes was investigated by a Hitachi S-4300 CFE SEM instrument. Prior to imaging, the specimens were coated with gold.

### 4.3. In Vivo Experimental Design

Adult male CD1 mice (5–6 weeks old) from our animal facility were used for this study. The experimental protocol was previously approved by the Ethics Commission of the Vasile Goldis Western University of Arad. Before and during the experiment, the mice were housed in IVC cages under conditions of constant temperature, humidity, and light/dark cycle and fed a standard diet.

The mice were randomly divided into 6 experimental groups (*n* = 10), as follows:

- Group 1 (control group) orally received a saline solution for 7 weeks and 0.7% carboxymethyl cellulose (CMC) for the next 2 weeks;
- Group 2 (CCl_4_ group) i.*p*. injected with CCl_4_ solution (20% v/v, 2 mL/kg b.w.) 2 times a week for 7 weeks and euthanatized for liver fibrosis confirmation;
- Group 3 (CCl_4_ control group) received CCl_4_ chemical induction of liver fibrosis for 7 weeks, followed by 2 weeks of de novo self-recovery (spontaneous fibrosis resolution);
- Group 4 (CCl_4_/CHR–RAMEB group) received CCl_4_ chemical induction of liver fibrosis for 7 weeks, followed by oral administration of 50 mg/kg CHR–RAMEB for 2 weeks;
- Group 5 (CCl_4_/CHR–HPBCD group) received CCl_4_ chemical induction of liver fibrosis for 7 weeks, followed by oral administration of 50 mg/kg CHR–HPBCD for 2 weeks;
- Group 6 (CCl_4_/CHR group) received CCl_4_ chemical induction of liver fibrosis for 7 weeks, followed by oral administration of 50 mg/kg free CHR for 2 weeks.

In the experiment, we chose the lowest dose of chrysin that was effective for the resolution of liver fibrosis in our previous studies [29,30].

All experimental groups, except for Group 2, were euthanized at the end of Week 9, and liver biopsies were processed for subsequent histological, immunohistochemical, and electron microscopic analysis. Liver biopsies were also properly frozen for molecular biology analysis.

### 4.4. Histology

Liver biopsies were fixed in a 4% formaldehyde solution in PBS and embedded in paraffin. Sections were stained with Fouchet van Gieson (Bio-Optica, Milano, Italy). Histological analysis was done using an Olympus BX43 microscope. Fibrosis was graded (according to the previously used protocol [36]) from Grade 0 (normal) to Grade 4 (severe fibrosis). Each sample was observed at 20× magnification. The degree of liver damage was expressed as the mean of 10 fields of view on each slide.

### 4.5. Immunohistochemistry

The liver sections were deparaffinized, rehydrated, and incubated overnight at 4 °C with the primary antibodies NF-κB, TNF-α, TGF-β1, Smad 2/3, and α-SMA (1:100 dilution). Immunodetection was performed using a polymer detection system (Novolink Max Polymer, Leica Biosystems, Wetzlar, Germany) and 3,30-diaminobenzidine (DAB) as a chromogenic substrate. The nuclei were stained with hematoxylin. Images were gained by light microscopy (Olympus BX43, Hamburg, Germany).

The intensity of Smad 2/3 immunopositivity was analyzed with ImageJ (64-bit) software. Five fields were selected randomly from each liver section. The results are presented as the percentage of brown-stained Smad 2/3-positive fields compared with the control group (set to 100%).

The staining evaluation criteria for NF-kB was performed based on the differentiation differences between brown (positive) and blue (counter) staining, according to the methods used previously [56,57]. Because NF-kB exerts its regulatory activity after translocation to the nucleus, only nuclear staining was counted. The percentage of positive cells was counted in 3 representative high-power fields per liver section. The percentage of te positive cells was recorded for each individual/experimental group.

### 4.6. Transmission Electron Microscopy

Liver samples were prefixed in a 2.7% glutaraldehyde solution in a 0.1 M phosphate buffer, washed in a 0.15 M phosphate buffer (pH 7.2), post-fixed in a 2% osmic acid solution in a 0.15 M phosphate buffer, dehydrated in acetone, and then embedded in the epoxy embedding resin Epon 812. Next, 70 nm sections were double-contrasted with uranyl acetate and lead citrate, and analyzed with a Tecnai 12 Biotwin TEM (Fei Company, Hillsboro, OR, USA).

### 4.7. Quantitative Real-Time PCR Analysis

Real-time quantitative polymerase chain reaction (qPCR) was applied to assess mRNA expression of TGF-β1, Smad 2/3, Smad 7, Col I, TIMP-1, α-SMA, MMP-2, MMP-3, MMP-9, TNF-α, IL-6, NF-kB p65, and NF-kB p50. Total RNA was extracted using the SV Total RNA isolation kit (Promega) and the quantity and quality were assessed using a spectrophotometer (NanoDrop 8000, Thermo Scientific, Waltham, MA, USA), then the revers transcription was done using the First Strand cDNA synthesis kit (Thermo Scientific, Waltham, MA, USA). RT-PCR was performed using the Maxima SYBR Green/ROX qPCR master mix (Life Technologies, Carlsbad, CA, USA) with an Mx3000PTM RT-PCR system. All samples were run in triplicate. The primers are shown in Table 1. Glyceraldehyde 3-phosphate dehydrogenase (GAPDH) was used as a reference gene and was assessed under the same experimental protocol. Relative expression changes were determined using the 2∆∆C(T) method [58].

### 4.8. MiRNA PCR Array Analysis

The expression of miRNA molecules involved in fibrosis was evaluated using a miScript PCR system (Qiagen). Total RNA was isolated from liver samples using the miRNeasy mini kit (Qiagen), which ensured the effective purification of miRNA and total RNA. The concentration and purity of RNA were determined using a NanoDrop 8000 spectrophotometer (Thermo Fisher Scientific, Waltham, MA, USA). RNA was reverse-transcribed to cDNA by the miScript II RT kit (Qiagen) using a Veriti 96-Well thermal cycler (Applied Biosystems, Foster City, CA, USA). The miRNA PCR array analysis was performed by the miScript SYBR green PCR kit (Qiagen) and the miScript miRNA PCR array for mouse fibrosis (Qiagen) using a ViiA 7 real-time PCR system (Thermo Fisher Scientific, Waltham, MA, USA). The PCR array contained primers for the detection of 84 miRNA molecules involved in fibrosis and 12 internal controls. The obtained data were analyzed using the GeneGlobe data analysis software provided by Qiagen.

### 4.9. Statistical Analysis

The statistical analysis was done using GraphPad Prism 3.03 software (GraphPad Software, Inc., La Jolla, CA, USA), one-way analysis of variance, and Bonferroni’s test; *p* < 0.05 was considered to indicate a statistically significant difference.

## 5. Conclusions

In conclusion, we determined that both CHR–HPBCD and CHR–RAMEB nanocomplexes exerted anti-inflammatory effects, decreased extracellular matrix accumulation and collagen, and improved the fibrotic structural and ultrastructural aspects of the liver. The beneficial effects were considerably higher than those for free chrysin administration. Mechanistically, antifibrotic and anti-inflammatory effects may occur in vivo by inhibition of the NF-κB and TGF-β1/Smad signaling pathways and by the modulation of hepatic pro/antifibrotic miRNA.

These results suggest that the use of chrysin-loaded β-cyclodextrin nanoparticles is a viable option for the oral delivery of flavonoids, which are considered to be potential therapeutic candidates with applications in the treatment of liver fibrosis.

## Figures and Tables

**Table 1:** Primer sequences for RT-PCR.

Target	Sense	Antisense
NF-κB 50	5′ AGGAAGAAAATGGCGGAGTT 3′	5′ GCATAAGCTTCTGGCGTTTC 3′
NF-κB 65	5′ CTTGGCAACAGCACAGACC 3′	5′ GAGAAGTCCATGTCCGCAAT 3′
TNF-α	5′CTGTAGCCCACGTCGTAGC3′	5′ TTGAGATCCATGCCGTTG 3′
IL-6	5′AAAGAGTTGTGCAATGGCAATTCT3′	5′AAGTGCATCATCGTTGTTCATACA 3′
TGF-β1	5′ TTTGGAGCCTGGACACACAGTAC 3′	5′ TGTGTTGGTTGTAGAGGGCAAGGA 3′
α-SMA	5′ CCGACCGAATGCAGAAG GA 3′	5′ ACAGAGTATTTGCGCTCCGAA 3′
Smad 2	5′ GTTCCTGCCTTTGCTGAGAC 3′	5′ TCTCTTTGCCAGGAATGCTT 3′
Smad 3	5′ TGCTGGTGACTGGATAGCAG 3′	5′ CTCCTTGGAAGGTGCTGAAG 3′
Smad 7	5′ GCTCACGCACTCGGTGCTCA 3′	5′CCAGGCTCCAGAAGAAGTTG 3′
Col I	5′CAGCCGCTTCACCTACAGC 3′	5′ TTTTGTATTCAATCACTGTCTTGCC 3′
TIMP-1	5′GGTGTGCACAGTGTTTCCCTGTTT 3′	5′ TCCGTCCACAAACAGTGAGTGTCA 3′
MMP-1	5′ GCAGCGTCAAGTTTAACTGGAA 3′	5′ AACTACATTTAGGGGAGAGGTGT 3′
MMP-2	5′CAGGGAATGAGTACTGGGTCTATT 3′	5′ ACTCCAGTTAAAGGCAGCATCTAC 3′
MMP-3	5′ACCAACCTATTCCTGGTTGCTGCT 3′	5′ATGGAAACGGGACAAGTCTGTGGA 3′
MMP-9	5′ GGACCCGAAGCGGACATTG 3′	5′ CGTCGTCGAAATGGGCATCT 3′
GAPDH	5′CGACTTCAACAGCAACTCCCACTCT3′	5′TGGGTGGTCCAGGGTTTCTTACTCCT3′

## Data Availability

The data presented in this study are available on request from the corresponding author. The data are not publicly available due to privacy.
